# Can high-cost spending in the community signal admission to hospital? A dynamic modeling study for urgent and elective cardiovascular patients

**DOI:** 10.1186/s12913-018-3639-z

**Published:** 2018-11-15

**Authors:** Deborah Cohen, Walter P. Wodchis, Andrew Calzavara

**Affiliations:** 1 University of Toronto, Institute of Health Policy, Management & Evaluation, Toronto, ON Canada; 2 Institute for Clinical Evaluative Sciences, Toronto, Ontario Canada; 3 University of Ottawa, School of Epidemiology and Public Health, Ottawa, Ontario Canada; 4 Trillium Health Partners, Institute for Better Health, Toronto, Ontario Canada; 5 Canadian Institute for Health Information, Ottawa, ON Canada

**Keywords:** High-cost, Healthcare spending, Community care, Acute care, Cardiovascular disease, Survival analysis

## Abstract

**Background:**

Much of the research on high-cost patients in healthcare has taken a static approach to studying what is actually a dynamic process. High-cost patients often utilize services across multiple sectors along care pathways, but due to the cross-sectional nature of many study designs, we lack a clear understanding of the temporal relationship between high-cost spending in community and acute care. Studying care trajectories for high cost patients with cardiovascular disease (CVD) can shed light on the dynamic interplay between community-based and acute care along the care continuum, and provide information about signals in community care that may indicate future elective and urgent hospitalizations.

**Methods:**

Using linked health administrative data in Ontario, Canada, 74,683 incident cases with cardiovascular disease were identified between the years 2009 and 2011. Patients were followed for 36 months (total study duration 2009–2014) until the first urgent or elective admission to hospital for a heart-related condition. We used an extended Cox survival model with competing risks to study the relationship between high-cost spending in community care with two mutually exclusive outcomes: urgent or elective hospitalizations.

**Results:**

Elective hospitalizations were most clearly signaled by a high-cost utilization of community-based specialist services in the month prior to hospital admission (hazard ratio 9.074, *p* < 0.0001), while urgent hospitalizations were signaled by high cost usage across all community-based sectors of care (from general practitioner & specialist visits, home care, laboratory services and emergency department (ED) usage). Urgent hospitalizations were most clearly signaled by high cost usage in ED in the month prior to hospital admission (hazard ratio 2.563, *p* < 0.0001).

**Conclusion:**

By studying the dynamic nature of patient care trajectories, we may use community-based spending patterns as signals in the system that can point to future and elective hospitalizations for CVD. These community-based spending signals may be useful for identifying opportunities for intervention along the care trajectory, particularly for urgent CVD patients for whom future hospitalizations are difficult to anticipate.

## Background

Much of the research on high-cost patients in healthcare has taken a static approach to studying what is actually a dynamic process [2, 8, 13, 33]. High-cost patients often utilize services across multiple sectors along care pathways [11, 37], but due to the cross-sectional nature of many study designs, we lack a clear understanding of the temporal relationship between high- cost spending in community and acute care. Health system reform efforts generally operate under the assumption that well integrated community-based care can serve to reduce expensive hospital visits [22, 25, 27], however the dynamic nature of this relationship is not well understood. Does high-cost utilization of community-based services serve as a protective function in reducing hospital admissions, or does high-cost utilization in the community signal an increased risk of hospitalization? Studies that examine these questions can shed light on the way that community based services may signal difficult-to-predict hospitalizations and advance the research by pointing to areas along the care trajectory where medical interventions could reduce the likelihood of future avoidable hospitalizations [16, 17, 26, 32].

At 7 billion dollars in direct healthcare costs per year, cardiovascular disease (CVD) represents one of the most costly diseases for the Canadian healthcare system [7, 29, 30]. CVD patients often have complex needs and utilize general practitioner (GP) services, specialists, home care, lab services and emergency departments (ED) within the community accordingly [12, 34, 37]. Community-based care pathways for CVD patients are often dependent upon whether their hospital admission type is urgent or elective [9]. Elective hospitalizations, which include non-emergency procedures to improve blood flow or heart function [3, 36], can be scheduled according to patient and physician convenience [10]. Elective patients are generally expected to encounter preplanned community-based diagnostic tests and examination procedures in the months prior to the hospital admission [28]. Less in known however, about community-based utilization patterns prior to urgent hospitalizations, where unpredicted level of illness and urgent timelines may limit the opportunities for scheduling community-based encounters prior to hospitalization [9]. The purpose of this study was two-fold: 1) to examine the general patterns of high cost utilization in the community for urgent and elective CVD care trajectories, and 2) to assess the dynamic relationship between high-cost spending in the community as an indicator of urgent and elective acute care admissions in a cohort of CVD patients in Ontario, Canada.

## Methods

### Cohort

This retrospective study used linked health administrative databases to identify the complete population of newly diagnosed CVD patients (2009 to 2011) between the ages of 40 and 105. Patients were required to be residents of Ontario, have a valid health insurance number, and be identified with a first time CVD diagnosis for at least of one the following conditions: acute myocardial infarction, cardiac arrhythmia, chronic heart failure, stoke or chronic coronary syndrome (see [20] for the validated set of International Classication of Disease (ICD-10) codes and Ontario Health Insurance Plan (OHIP) diagnosis codes that were used to identify these CVD conditions). To be identified as a case in the cohort, patients had to have at least one acute hospitalization and/or at least two diagnoses recorded in doctor’s offices within the 2 year period. If one of these criteria was satisfied, the earliest healthcare encounter date became the time of index CVD diagnosis [5, 14, 15, 20, 23]. Incident cases were established by examining previous diagnosis codes in OHIP and hospital discharge records for up to 10 years prior to the index CVD event. Patients were followed for up to 36 months from index diagnosis with a study end date of March 2014.

### Data sources

Ontario health administration data housed at the Institute for Clinical Evaluative Sciences (ICES) were used in this analysis. Data were linked anonymously using unique encoded identifiers at the patient level. The Canadian Institute for Health Information (CIHI) Discharge Abstract Database (CIHI DAD) contains all hospital discharges in Ontario. The OHIP billing database contains all community-based healthcare services including physician visits and laboratory services. The Home Care Database contains all home care visits in Ontario. The CIHI National Ambulatory Care Reporting System contains all emergency department visits in Ontario [18]. The Registered Persons Database was used to identify patient demographics and patient deaths [18]. The Johns Hopkins ACG® System Version 10 was used to determine a comorbidity score.

### Statistical approach

Time-to-event survival modelling was used to examine the way in which high cost community-based spending for GP care, specialist care, home care, laboratory services and ED care were predictors of urgent and elective hospital admissions for CVD along the care pathway.

#### Outcome measure

The outcome measure of interest was the time to a heart-related hospital admission (either urgent or elective), following the index event diagnosis. The date of admission to hospital served as the date at which the outcome occurred.

Independent Measures: Survival models contained time constant and time-varying covariates. Time constant variables included patient rural/urban residence, patient socioeconomic status (area based measure) [24], an ACG® System Aggregated Diagnosis Groups (ADGs) comorbidity score [19] equal to the sum of the ADGs, and a set of baseline comorbidities commonly associated with cardiovascular disease including diabetes, hypertension, chronic obstructive pulmonary disease, renal failure and mood disorders [4, 20]. Time-varying covariates included monthly community-based costs for GP visits, specialist visits, home care and laboratory services and ED visits. Costs for each of these community-based services, discounted to the nominal base year, were calculated for each month of follow up based on the %getcost macro developed at ICES [37]. High cost utilization of community care was identified at the 95th percentile of total monthly spending for each category of care: GP physician visits, specialist visits, home care, laboratory services and ED visits. Patients were coded as having a high-cost utilization (coded as a binary variable (0 – low cost, 1 – high cost) in each month of follow up along the care trajectory until the hospitalization event occurred, the patient died or the follow up period ended.

#### Dynamic models

An extended Cox survival model using competing risks was used to conduct the time-to-event analysis with time-constant and time-varying covariates [1]. A competing risk model allowed for the examination of two mutually exclusive hospitalization outcomes: urgent or elective hospitalization admissions. A 1 month time lag between community-based spending and hospitalization was built into the analysis in order to correct for potential reverse causation [1, 35]. Patients who died within their follow up window were right censored. All independent variables were assessed using a univariate chi-square analysis for inclusion in the model (*p* < 0.01). The proportional hazards assumption was tested using Schoenfeld residuals. Age group and sex were found to violate proportionality [1] however sensitivity testing showed consistent patterns between community-based spending and hospitalization across the age and sex groups for both urgent and elective hospitalizations. Other sensitivity testing included rerunning the models with a 2 month lag, and a one and 2 month lag combined. In all cases, the 1 month lag which was selected as the time varying approach in the final model had the strongest association to the outcome. Ties were dealt with using the Effron tied method [1]. Hazard ratios were used to measure the instantaneous rate of hospital admission at any time (t) along the patient trajectory [31]. The use of data in this project was authorized under section 45 of Ontario’s Personal Health Information Protection Act.

## Results

Table 1 presents patient characteristics for the complete cohort and for patients with urgent and elective hospitalization outcomes. A total of 74,683 patients were included in the analysis. With an average age of 67 years, 52% of the patients were male, and 10% lived in a rural community. Most patients were part of a primary care model associated with a health team rather than solo-practice physician (79%), the cohort had an average ADG score of 7.5 and 15% died during the follow-up period. The majority of patients had no hospitalization event during the follow up period (75%), 2% had an elective hospitalization and 8% had an urgent hospitalization. The proportion of CVD specific diseases and co-morbid illness were consistent with previous research [12, 20]. Chronic coronary syndrome and arrhythmias were most common at 46% and 31% respectively. Hypertension and diabetes were the most common comorbidities at 65% and 27% respectively.

**Table 1:** Patient Characteristics – Overall Cohort and Patients with Urgent and Elective CVD-related Hospitalization Outcomes

	Overall Cohort (*n* = 74,683)	Patients with Elective CVD-related Hospitalization Outcome (*n* = 1482)	Patients with Urgent CVD-related Hospitalization Outcome (*n* = 6047)	Chi-Square *p* Value
Characteristics	% (n)			
% rural	9.52 (7110)	11.20 (166)	11.15 (674)	0.9519
% male	51.76 (38655)	75.17 (1114)	51.91 (3139)	< 0.0001
% senior	54.75 (40886)	48.58 (720)	71.14 (4302)	< 0.0001
Average age	66.63 (SD 13.15)	64.47 (SD 10.35)	71.96 (SD 12.77)	< 0.0001
Patient income				0.0020
% high income	20.32 (15172)	20.51 (304)	18.34 (1109)	
% low income	19.52 (14580)	18.83 (279)	22.39 (1354)	
Average ADG score	7.47 (SD 3.77)	6.67 (SD 3.47)	7.81 (3.87)	< 0.0001
Primary Care Model				
FFS	16.35 (12207)	16.60 (246)	17.05	0.7268
Health Team	78.68 (58758)	78.68 (1166)	77.31 (4675)	
Other	4.98 (3718)	4.27 (70)	5.64 (341)	
Patient Outcomes				
% died during follow-up	15.23 (11371)	–	–	
% elective hospitalization	1.98 (1482)	–	–	
% urgent hospitalization	8.09 (6047)	–	–	
% no event	74.63 (55738)	–	–	
Baseline CVD condition				
AMI	14.38 (10736)	13.02 (193)	21.04 (1272)	< 0.0001
Arrhythmia	30.90 (23076)	18.89 (280)	31.83 (1925)	< 0.0001
CHF	18.15 (13554)	14.91 (221)	32.89 (1989)	< 0.0001
Coronary Syndrome	46.01 (34364)	80.31 (1190)	45.82 (2771)	< 0.0001
Stroke	13.63 (10180)	4.39 (65)	14.98 (906)	< 0.0001
Baseline Co-morbidities				
Diabetes	26.89 (20085)	37.18 (551)	38.04 (2300)	0.5427
Hypertension	65.62 (49010)	67.21 (996)	78.02 (4718)	< 0.0001
COPD	10.01 (7477)	7.29 (108)	15.25 (922)	< 0.0001
Renal Failure	3.83 (2858)	3.44 (51)	7.54 (456)	< 0.0001
Mood Disorders	16.79 (12541)	10.53 (156)	14.32 (866)	0.0001

*CVD* Cardiovascular disease, *AMI* Acute myocardial infarction, *ADG* Aggregated diagnosis groups, *FFS* Fee for service

Urgent patients were generally older and sicker than elective patients (with average ages of 72 versus 64 years respectively, *p* < 0.0001). Urgent patients had more comorbidities than elective patients (average ADG score 7.8 versus 6.7 respectively, *P* < 0.0001), had higher rates of every baseline CVD condition (with the exception of coronary syndrome), and higher rates of specific baseline comorbidities.

Table 2 presents monthly cost distributions for each of the five community-based care categories of spending: GP, specialist, home care, laboratory and ED. All cost distributions were highly right skewed. In the case of ED, home care and lab services, the distributions were also zero-inflated, as many patients did not incur a cost in each of these healthcare categories every month. As a result of these zero-inflated, highly skewed distributions, costs at the 95th percentile were selected to indicate a high-cost month. High cost spending thresholds were set at any monthly patient cost exceeding $147 for GP care, $584 for specialist care, $274 for home care, $87 for laboratory services, and $115 for ED care based on monthly costing distributions (Canadian dollars).

**Table 2:** Monthly Patient Costs for Community-Based Services across the Care Trajectory

Monthly Cost Distributions	Range ($)	50th percentile	95th percentile
GP Care	(0–9777.33)	$24.18	$147.36/month
Specialist care	(0–25,661.22)	$ 0	$584.20/month
Home Care	(0–32,028.35)	$ 0	$274.07/month
Laboratory	(0–1091.72)	$ 0	$87.15/month
Emergency Department	(0–8234.70)	$ 0	$114.74/month

**Fig. 1:**
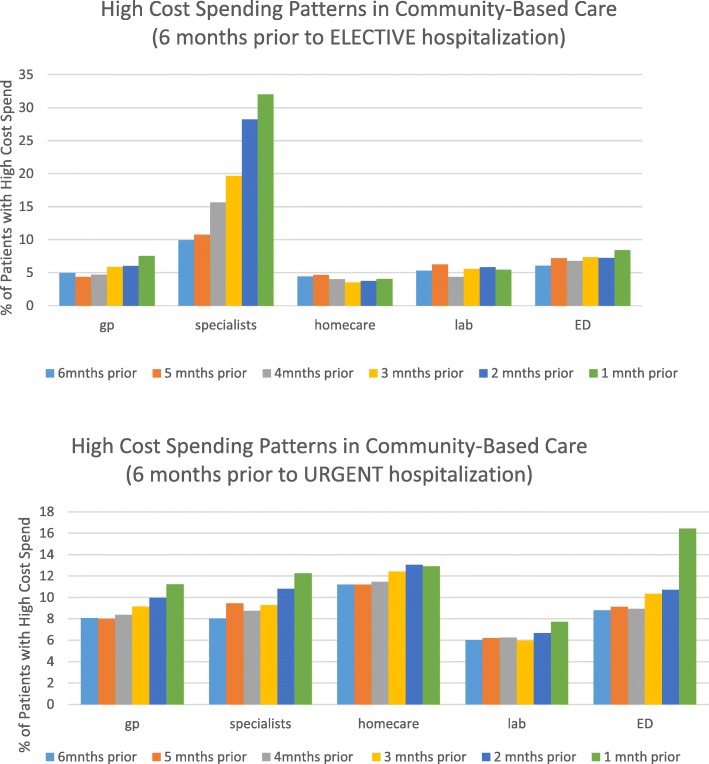
High cost utilization in the 6 months preceding the hospital admission

Figure 1 presents the high cost spending patterns in community-based care for 6 months prior to the elective or urgent hospitalizations. The graph demonstrates a general increase in high- cost spending across all community-based sectors in the months leading up to the hospitalization, however elective and urgent care trajectories had several important distinctions. Elective trajectories had a stark signal related to high-cost specialist use in the month prior to hospitalization. Alternatively, urgent trajectories showed a more consistent high cost utilization pattern across all forms of community-based care. For urgent cases, the most apparent high-cost signal was in ED use in the month prior to hospitalization.

Table 3 presents the results of the extended Cox survival models for community-based spending as time-varying predictors of urgent and elective hospitalizations. In the case of elective hospitalizations, high-cost spending in specialist care, home care and ED care in were significant predictors of hospitalization, while high-cost spending in GP care and laboratory services were not. For elective hospitalizations, the clearest signal in the system was high-cost specialist care with a hazard ratio of 9.07 (*p* < 0.0001). Home care and ED actually appeared to have a small but significant protective effect with hazard ratios at 0.55 (*p* = 0.0002) and 0.78 (*p* = 0.0246) respectively.

**Table 3:** Extended Survival Models for High Cost Community-Based Care: Primary Care, Specialist, Home Care, Laboratory Services and Emergency Department Care as Time Varying Predictors of Hospital Admission

	Beta	SE	Chi-square	P	HR
Survival Model – ELECTIVE Hospitalization					
Monthly High cost GP	−0.12121	0.10581	1.3123	0.2520	0.886
Monthly High cost Specialist	2.20546	0.05964	1367.4911	< 0.0001	9.074
Monthly High cost homecare	−0.59350	0.16195	13.4294	0.0002	0.552
Monthly High cost laboratory	−0.04933	0.11268	0.1916	0.6616	0.952
Monthly High cost emergency department	−0.24214	0.10776	5.0490	0.0246	0.785
Survival Model – URGENT Hospitalization					
Monthly High cost GP	0.14998	0.04485	11.185	0.0008	1.162
Monthly High cost Specialist	0.3736	0.04485	72.3877	< 0.0001	1.456
Monthly High cost homecare	0.32515	0.04056	64.2785	< 0.0001	1.384
Monthly High cost Laboratory	0.18537	0.04889	14.3784	< 0.0001	1.204
Monthly High cost emergency department	0.94101	0.03895	583.8090	< 0.0001	2.563

Other variables in the models included sex, age, rural/urban, ADG score, SES, GP Care Model (FFS, Family Health Team, Other), baseline CVD condition (AMI, arrhythmia, CHF, coronary syndrome, stroke), and baseline co-morbidities (diabetes, hypertension, COPD, renal failure, mood disorders)

Conversely, for urgent hospitalizations, the high-cost signals in community-based spending were significant for all sectors of spending but they were much more equally distributed across all sectors of community care. High-cost emergency department usage had the largest signal with a hazard ratio of 2.56 (*p* < 0.0001), while high-cost GP, specialist, home care and lab usage had smaller but significant hazard ratios (at 1.16 (*p* = 0.0008), 1.46 (p < 0.0001), 1.38 (*p* < 0.0001) and 1.20 (*p* < 0.0001) respectively).

## Discussion

The dynamic relationship between community and acute sectors of care is a growing part of the ‘integrated care’ policy discourse focused on the reduction of health system costs, the delivery quality care and the improvement of patient health outcomes [6]. Studies that examine high-cost use within healthcare at a point in time can fail to grasp the temporal nature of high-cost use and the way that community-based care may be leveraged to help signal and prevent future high-cost hospitalizations. This study demonstrated that there were distinct patterns of community-based high-cost care for CVD patients that were dependent upon elective or urgent care trajectories. Elective hospitalizations were most clearly signaled by a high-cost utilization of community-based specialist services in the month prior to hospital admission, while urgent hospitalizations were signaled by high-cost usage across all community-based care, with a specific signal in high cost utilization of ED. By studying the dynamic nature of patient care trajectories, it is possible that these community-based spending patterns could serve as signals in the system that could point to opportunities for intervention along the CVD care trajectory. For example, regular tracking of high cost ED spending by health systems planners and managers could be used to identify patients requiring more rigorous home care visits and primary care follow up, in order to intervene along the care pathway and reduce potentially avoidable future urgent care admissions [16].

According to discharge abstract coding standards, elective hospitalizations refer to admissions for interventions that are planned for and scheduled in advance of the hospital admission [10]. .Elective procedures for CVD range from minimally invasive (e.g. angioplasty) to very invasive (e.g. coronary artery bypass grafting), but in every case there is a planned and anticipated link between the clinical level of need and the corresponding level of care/treatment [21, 28]. In these cases, practice guidelines dictate an expected higher use of specialist services in the community in preparation for the elective hospital procedure [3, 28, 36]. The results of this analysis confirmed this relationship, finding that for elective patients, high-cost visits to specialists were a clear signal in the system with a hazard ratio of 9.07. Although this analysis did not drill down into the types of services that make up the high cost specialist visits, as a macro-level signal, spending on specialists appeared to be consistent with anticipated patterns between community-based care and acute care elective CVD patients.

One the other hand, urgent care patients present a more complex problem. By definition, an urgent hospitalization occurs when a patient is admitted for a life-threatening condition and/or requires immediate treatment that cannot be accommodated or anticipated by a scheduled visit [10]. .Urgent patients have traditionally presented a more significant planning challenge for the healthcare system because the clinical need and corresponding treatment is often unanticipated and can therefore not be scheduled [9]. In this case, health system manager’s assessments of high-cost spending signals in the community may be particularly informative to help anticipate and prevent future urgent hospitalizations. Furthermore, this knowledge may point to opportunities to intervene along the patient care trajectory in order to reduce or mitigate the need for future urgent hospitalizations altogether. The results of this analysis demonstrated that high-cost spending in virtually every sector of community-based care served as a significant signal for increased the rate of urgent hospitalization. High-cost spending in ED was the strongest signal in community care, flagging that CVD patients with a high-cost ED usage had 2.6 times the instantaneous rate of urgent hospitalization in the following month. Although specific services utilized in ED were not assessed, these findings point to a critical macro level signal - high cost ED usage - as an important predictor of urgent CVD hospitalization. Using this baseline information, future research that considers which interventions at this point along the care trajectory (either within the ED itself or in triggering follow up GP care, pharmaceutical care or others) may best result in avoiding future preventable hospitalizations.

A number of limitations warrant consideration. First, data on pharmaceutical spending was not available for most individuals under the age of 65 and therefore was not included in this analysis. As an important part of community care for CVD, from prevention to disease management, future research should study high cost CVD pharmaceutical use as a signal as well as an important potential system intervention. Second, given that this study used an incident cohort of CVD patients, this study may not be generalizable to the prevalence-based population of CVD patients. Third, we opted to examine high-cost signals in community-based care with a 95% threshold cut-off. There may be other ways that spending in community care that would create an even more robust signal. It is most certainly that case that the more narrow the time span for the unit of analysis (we used a monthly unit of analysis in this case), the more precise the signals can become. Future work should examine other ways, both in terms of spending cut-offs and time units of analysis, to model spending signals in the community. Fourth, it is important to note that hazard ratios for extended cox models with competing risks must be interpreted as instantaneous rates and not as instantaneous hazards [31]. And finally high-cost spending, as a proxy for overall intensity of use, represents a macro-level systems approach to studying the dynamic interplay in community and acute care along the continuum. However, specific CVD services (e.g. treatments, diagnostic tests, etc.) that may be important signals were not within the scope of this study. Future work that examines the role of specific services in the community as key predictors of future hospitalizations is also warranted.

## Conclusion

High-cost care trajectories for CVD patients can shed light on the dynamic nature of the continuum of care, providing important information about signals in community care that may best indicate elective and urgent hospitalizations. This study adds considerable specificity to existing research that broadly characterizes high-cost, high-needs patients [37] by grounding measurement of appropriate outcomes in a clinically defined population. This type of information is particularly useful for urgent CVD patients for whom future hospitalizations are difficult to plan for and anticipate.

## Abbreviations

ADG: Aggregated diagnosis groups
CIHI DAD: Canadian Institute for Health Information Discharge Abstract Database
CVD: Cardiovascular disease
ED: Emergency department
GP: General practitioner
ICD: International classification of disease
ICES: Institute for Clinical Evaluative Sciences
OHIP: Ontario Health Insurance Plan
